# Magnetic Resonance Imaging Segmentation of Soft Tissue in the Diagnosis of Chronic Low Back Pain: A Scoping Review

**DOI:** 10.3390/diagnostics16121832

**Published:** 2026-06-13

**Authors:** Wiktoria Frącz, Anita Bilska, Jakub Matuska, Pablo Herrero, Elżbieta Skorupska

**Affiliations:** 1 Department of Physiotherapy, Poznan University of Medical Sciences, 61-702 Poznan, Poland; abilska@ump.edu.pl (A.B.); jakub.matuska@student.ump.edu.pl (J.M.); skorupska@ump.edu.pl (E.S.); 2 Department of Physiatry and Nursing, Faculty of Health Sciences, University of Zaragoza, 50009 Zaragoza, Spain; pherrero@unizar.es; 3 Unit of Histology and Neurobiology, Department of Basic Medical Sciences, Faculty of Medicine and Health Sciences, Rovira I Virgili University, 43003 Reus, Spain; 4 iHealthy Research Group, Instituto de Investigación Sanitaria (IIS) Aragon, HCU Lozano Blesa, 50009 Zaragoza, Spain

**Keywords:** cross-sectional area, fatty infiltration, muscle atrophy, muscle composition, lumbosacral region, back muscles, biomarkers, pain measurement, muscle segmentation, diagnostic imaging

## Abstract

**Background/Objectives**: Despite the substantial clinical and socioeconomic burden of chronic low back pain (CLBP), objective diagnostic biomarkers remain limited. Structural alterations of the lumbosacral muscles, particularly muscle atrophy and fatty infiltration (FI), have been proposed as imaging correlates of chronic pain. This scoping review aimed to synthesize current evidence on these alterations in CLBP and characterize the imaging and segmentation methods used. **Methods**: The review was conducted in accordance with PRISMA-ScR guidelines and guided by the Population–Concept–Context framework. Population: adults with CLBP. Concept: MRI segmentation techniques are used to evaluate morphological soft-tissue changes. Context: clinical and research settings using MRI for CLBP evaluation. A comprehensive search of PubMed, Scopus, Embase, and Web of Science was performed for studies published between January 2014 and October 2024. **Results**: Twelve observational studies met the inclusion criteria. Degenerative alterations were consistently observed in CLBP and were not reported in control groups. Muscle atrophy was reported in ten studies (multifidus [MF]: 9; erector spinae [ES]: 7; psoas major [PM]: 2; paraspinal muscles [PPM]: 1; and increased FI in all studies (MF: 9; ES: 5; PM: 2; PPM: 2). Considerable heterogeneity between studies was noted. **Conclusions**: Lumbosacral muscles assessment may provide useful objective information for a more objective characterization of CLBP. Degenerative alterations were reported in all examined muscles except QL, with the MF most consistently affected. Changes in ES and PM may be specific for CLBP. The frequent co-occurrence of muscle atrophy and FI suggests that their combined evaluation may provide complementary information.

## 1. Introduction

Low back pain (LBP) is the most common musculoskeletal disorder and a leading cause of disability worldwide. In 2020, about 619 million people were affected by LBP, with projections reaching 843 million by 2050 [1]. Chronic low back pain (CLBP) represents at least 90% of LBP cases in primary care [2]. Since chronic pain has been recognized as a distinct disease entity in ICD-11, there is a clear need to develop new approaches to its management.

Despite its high prevalence and clinical burden, there is still a lack of consensus on effective diagnostic strategies for patients with CLBP. This has led to an increasing interest in identifying quantitative biomarkers that could support diagnosis and patient stratification. One promising approach involves assessing soft-tissue alterations as contributing factors to chronic pain, particularly in the form of muscle degeneration in the lumbosacral area. These degenerative changes include muscle atrophy, which is described as a progressive loss of muscle mass that impairs functional performance [3], and increased fatty infiltration (FI), defined as the abnormal accumulation of lipids within and around muscle tissue. Both factors are considered markers of reduced muscle quality, and their presence has been reported in individuals with CLBP along with decreased muscle function [4,5,6].

The literature indicates that muscles of the lumbosacral area, such as multifidus (MF), erector spinae (ES), psoas major (PM) and quadratus lumborum (QL), may undergo morphological alterations in individuals with CLBP [7,8,9]. However, it remains unclear which of these muscles have been most frequently investigated, which outcomes are most commonly reported, and whether these alterations are typically analyzed in isolation or in combination. It is also uncertain whether studies emphasize one of these features over the other or consider them jointly as complementary indicators of muscle quality.

Longstanding soft-tissue alterations in total muscle volume (TMV) and cross-sectional area (CSA) can be assessed using magnetic resonance imaging (MRI) [10,11,12]. Manual segmentation of MRI remains the gold standard for soft-tissue assessment, but semi-automated and fully automated methods have been increasingly explored to streamline the measurement process [13,14]. Scoping reviews are particularly suitable for mapping broad and heterogeneous bodies of literature, mainly when key concepts, methodologies, or outcome measures have not yet been consistently defined. In the context of muscle alterations in chronic low back pain, existing studies vary widely in terms of populations, imaging modalities, segmentation approaches, and outcome reporting, making a systematic synthesis unfeasible. We therefore conducted a scoping review to explore and categorize the available evidence on this topic comprehensively. To the best of our knowledge, this is the first scoping review specifically addressing soft-tissue changes in the lumbosacral region of patients with CLBP.

The main aim of this study is to map and synthesize the existing literature on the assessment of soft-tissue alterations, specifically muscle atrophy and fatty infiltration, in the lumbosacral area of patients with CLBP, and to explore their proposed role as potential imaging biomarkers. In addition, we examine the technical characteristics of the imaging and segmentation methods used for this purpose.

## 2. Materials and Methods

The scoping review was conducted and reported in accordance with the Preferred Reporting Items for Systematic reviews and Meta-Analyses extension for Scoping Reviews (PRISMA-ScR) guidelines [15] (File S1). The review protocol was not registered. This study was planned based on the PCC (Population, Concept, Context) framework [16] as follows: (P) Population: adults (>18 years) with self-reported or clinically diagnosed CLBP or LBP patients fulfilling chronic pain criteria; (C) Concept: application of MRI segmentation to evaluate morphological changes in soft tissues, with outcomes referring to muscle atrophy and fatty infiltration; (C) Context: clinical and research settings in which MRI is applied for the diagnosis and investigation of CLBP.

### 2.1. Search Methods for Identifying Studies

The following databases were evaluated: PubMed, Scopus, Embase, and Web of Science from January 2014 to October 2024. The search string included combinations of medical subject headings and abstracts relevant to population, concept, and context of the review. Only articles in English were considered. The strategy was developed in PubMed and adapted with appropriate changes for other databases (Table 1). On 15 October 2024, the final search was conducted to identify any newly published studies for inclusion before completing the manuscript. The literature search, study selection, data extraction, and quality assessment were performed by two independent reviewers.

### 2.2. Eligibility Criteria

We defined the following inclusion criteria: observational (cohort, case–control, or cross-sectional) studies written in English; participants aged ≥18 years with chronic low back pain (CLBP), either self-reported or diagnosed by a clinician through interview, physical examination, or imaging; studies investigating morphological changes in the lumbar multifidus, lumbar erector spinae, psoas major, or quadratus lumborum muscles; studies containing at least one of the following outcomes referring to muscle atrophy (cross-sectional area, relative cross-sectional area, relative muscle cross-sectional area, total cross-sectional area, functional cross-sectional area, muscle cross-sectional area, average cross-sectional area, total muscle volume), and at least one of the following outcomes referring to fatty infiltration (fat fraction, fat infiltration, intramuscular fat, signal intensity, proton density fat fraction); use of magnetic resonance imaging (MRI) to assess muscle morphology through segmentation.

We defined the following exclusion criteria: studies not including participants with CLBP; studies not including a control group consisting of healthy volunteers; studies not utilizing MRI segmentation; studies without cross-sectional area and/or volumetric assessment; preprints or not peer-reviewed.

### 2.3. Study Selection

Mendeley (version 2.145.0, Elsevier, Amsterdam, The Netherlands) was used for removing duplicates, initial screening, and full-text review. Two independent reviewers screened titles and abstracts, followed by full-text assessment of potentially eligible studies. Studies that did not meet the eligibility criteria were excluded from further analysis.

### 2.4. Data Extraction

Data extraction was performed independently by two reviewers using a standardized form designed for this scoping review. The following information was registered: year of publication, first author, study design, sample size, mean age (years), examined muscles, examined spinal level, MRI, assessment characteristics, segmentation method and results.

### 2.5. Risk-of-Bias and Publication Bias Assessment

In accordance with scoping review methodology, no critical appraisal of individual studies was performed, as the aim of this review was to map and describe the existing evidence rather than to evaluate study quality.

## 3. Results

### 3.1. Literature Search and Selection of Studies

**Figure 1:**
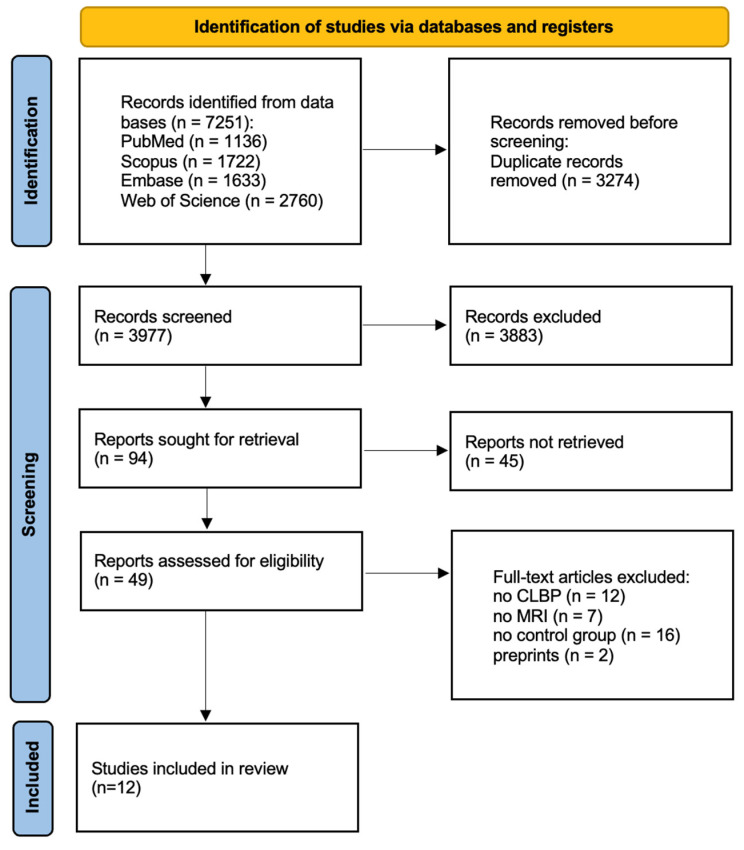
The PRISMA 2020 flow diagram for identification of studies via databases and registers [17].

The total number of studies found in screened databases was 7251: 1136 in PubMed, 1722 in Scopus, 1633 in Embase, and 2760 in Web of Science. After removing duplicates, 3977 articles were screened based on titles and abstracts. A total of 49 articles were included for full-text review, and after final screening, 12 studies were included in this review. The PRISMA 2020 flow diagram presents the selection process of the studies (Figure 1).

### 3.2. Assessment of Muscles in the Lumbosacral Area (Atrophy and Increased FI) as a Biomarker for CLBP

A total of twelve observational studies investigating morphological changes in the lumbosacral muscles of CLBP patients were included in the analysis. The most frequently investigated muscle was multifidus (*n* = 12), next erector spinae (*n* = 9) and psoas major (*n* = 9), and lastly quadratus lumborum (*n* = 2). In two studies, the MF, ES, and PM muscles were grouped and assessed collectively as paraspinal muscles (PPM). Muscle selection and grouping varied across studies, with different anatomical configurations explored (see Table 2).

The studies varied in terms of the anatomical levels assessed, with nine studies evaluating multiple lumbar levels and three studies focusing on a single level assessment, ranging from L1 to S1. Sample sizes varied considerably, ranging from *n* = 30 to *n* = 1681 for patients meeting CLBP criteria, and from *n* = 15 to *n* = 6953 for healthy volunteers. In addition to CLBP cohorts, some studies also included other comparison groups, such as patients with acute LBP (*n* = 2), non-CLBP patients (*n* = 1), and individuals with lumbar disc herniation without LBP symptoms (*n* = 1). Most studies examined the middle-aged and older population, whereas a few studies examined the younger adult population (Table 2). Detailed characteristics of the included CLBP populations are presented in Supplementary Table S1 (File S1).

Six studies utilized 1.5 Tesla (T) MRI, four 3 T MRI and in two studies, the MRI field strength could not be determined. The included studies used a variety of MRI sequences, such as T1, T2, and Dixon-based acquisitions. Bilateral measurements were used in most studies (*n* = 10), one study employed unilateral measurement, and in one case, it was not specified whether the measurement was bilateral or unilateral. Segmentation methods were as follows: manual (*n* = 4), semi-automatic (*n* = 4), automatic (*n* = 1), and in three studies, this could not be verified. Semi-automated approaches typically combined manual muscle delineation with software-assisted image analysis (e.g., histogram-based quantification, threshold-based tissue classification, and 3D volumetric reconstruction) [18,19,20,21], whereas fully automated methods relied on deep-learning algorithms, particularly convolutional neural networks (CNNs) based on U-Net architectures [22]. Multiple metrics were used for quantifying muscle atrophy (e.g., decreased CSA) and FI, reflecting methodological variability across studies in how muscle degeneration was assessed (see Table 2).

**Figure 2:**
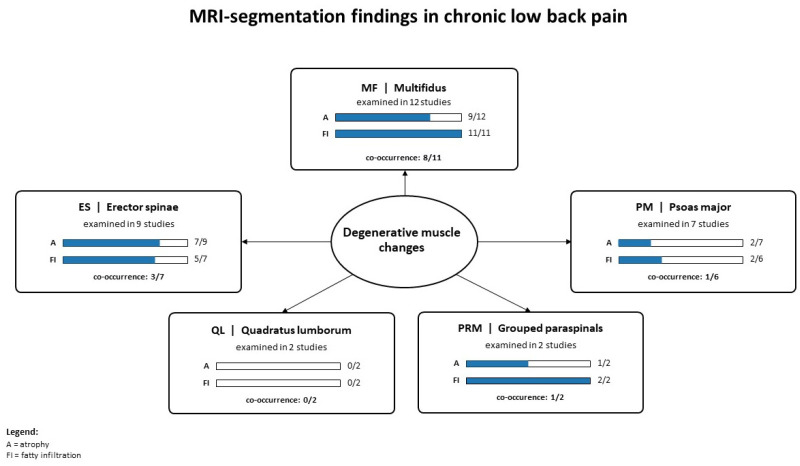
MRI-segmentation findings in chronic low back pain. Overview of MRI-derived muscle changes across studies, showing that atrophy and fatty infiltration were most consistently reported in the multifidus and erector spinae, with less frequent findings in psoas major, quadratus lumborum, and grouped paraspinal muscles.

Evidence of muscle atrophy among CLBP patients was reported in ten of the twelve included studies: nine in MF muscle, seven in ES muscle, two in PM muscle, one in PPM muscles (MF, ES and PM examined together), and zero in QL muscle. Increased FI among CLBP patients occurred in all studies: nine studies for MF, five studies for ES, two studies for PM, two studies for PPM, and zero studies for QL. Both degeneration changes were confirmed in: eight studies for MF, three studies for ES, and one study for PM (see Figure 2). Importantly, none of the included studies reported muscle degeneration changes in the healthy volunteer groups (see Table 2 and Table 3).

**Table 2:** Characteristics of the included studies examining CLBP in relation to muscle degeneration changes.

Study	Muscle	Level	Participant Characteristics	MRI Settings	Methods	Outcomes
Sions, 2016 [18]	MF	single level, L5	CLBP *n* = 57 age: 70.5 ± 6.8 Healthy *n* = 49 age: 72.2 ± 6.6	1.5 T, T1	bilateral measurements, semi-automatic: - atrophy: rCSA, rmCSA; - FI: MFI	No atrophy of MF in CLBP patients compared to healthy volunteers (*p* > 0.05). Increased FI of MF in CLBP patients compared to healthy volunteers (*p* = 0.034).
Huang, 2022 [23]	MF, ES	multilevel, L4–S1	CLBP *n* = 60 age: 46.3 ± 17.0 Healthy *n* = 32 age: 44.9 ± 17.6	3 T, T1 + T2 + IDEAL-IQ	bilateral measurements, manual segmentation: - atrophy: CSA; - FI: PDFF	Atrophy of MF at L4/5 in CLBP patients compared to healthy volunteers (*p* < 0.05). No atrophy of MF at L5/S1 and of ES at any examined level. Increased FI of MF and ES in CLBP patients compared to healthy volunteers (*p* < 0.05).
Sakai, 2017 [24]	MF, ES	multilevel, L1/2 and L4/5	CLBP *n* = 100 age: 74.4 ± 6.0 Healthy: *n* = 560 age: 73.2 ± 7.6	MRI, T2	bilateral measurements, - atrophy: CSA; - FI: FI	Atrophy of MF and ES at both levels in CLBP patients compared to healthy volunteers (*p* < 0.05), except for ES at L4/5 in males (*p* > 0.05). Increased FI of MF in CLBP patients compared to healthy volunteers (*p* < 0.01). FI of ES was not examined.
Fan, 2023 [25]	MF, ES	multilevel, L4–S1	CLBP *n* = 83 age: 29.9 ± 7.5 non-CLBP *n* = 32 age: 31.0 ± 6.6 Healthy *n* = 66 age: 27.8 ± 10.2	3 T, T1 + T2 + mDIXON-Quant	unilateral measurements (painful side or average of both sides if pain location is uncertain), manual segmentation: - atrophy: CSA; - FI: FF	Atrophy of MF at L4 upper endplate in CLBP patients compared to healthy volunteers (*p* = 0.013) and all 3 groups (*p* = 0.016). Atrophy of ES at L4 in CLBP patients compared to healthy volunteers (*p* = 0.008) and all 3 groups (*p* < 0.005). Increased FI of MF and ES in CLBP patients compared to healthy volunteers and all 3 groups (*p* < 0.05).
Chen, 2023 [26]	MF, PM	single level, L4–L5	LBP with LDH (pain duration: >3 months) *n* = 208 age: 43.4 ± 10.6 non-LBP LDH *n* = 104 age: 43.2 ± 11.0 Healthy *n* = 160 age: 44.2 ± 11.2	3 T, T1 + T2	segmentation: - atrophy: CSA, fCSA; - FI: Kjaer method and fatty degeneration	Atrophy of MF in LBP LDH patients compared to healthy volunteers (*p* < 0.05). Increased FI of MF in LBP LDH patients compared to non-LBP LDH patients (*p* = 0.046). Increased FI of MF in the healthy group compared to the investigated groups (*p* < 0.001). No associations of PM in LBP LDH patients compared to non-LBP LDH patients.
Zhu, 2023 [27]	MF, ES, PM	multilevel, L2–S1	CNLBP *n* = 58 age: 30.4 ± 3.9 Healthy *n* = 60 age: 29.3 ± 3.3	1.5 T, T2	bilateral measurements, manual segmentation - atrophy: CSA; - FI: higher mean MRI signal intensity	No atrophy in CLBP patients compared to healthy volunteers (*p* > 0.01). Increased FI of all examined muscles in CLBP patients compared to healthy volunteers (*p* < 0.01).
Alami, 2024 [19]	MF, ES, PM	multilevel, L2–S1	CNLBP with LSI *n* = 15 age: 34.4 ± 7.3, CNLBP without LSI *n* = 15 age: 38.4 ± 6.2 Healthy *n* = 15 age: 34.1 ± 4.9	1.5 T, T2	bilateral measurements, semi-automatic segmentation: - atrophy: CSA, rmCSA; - FI: MFI	Atrophy of MF at L3/L4 to L5/S1 in CLBP LSI (*p* = 0.042) and no LSI patients (*p* = 0.011) compared to healthy volunteers. Atrophy of ES at L4/L5 in CLBP LSI patients compared to healthy volunteers. (*p* = 0.044). Increased FI of MF at L3/L4 to L5/S1 in CLBP LSI (*p* = 0.034) and no LSI patients (*p* = 0.011) compared to healthy volunteers. No atrophy or increased FI associations were found in other muscles on any level.
Wesselink, 2024 [22]	MF, ES, PM	multilevel, L1–L5	CLBP *n* = 1681 age: 63.7 ± 7.5 aLBP *n* = 930 age: 62.5 ± 7.6 Healthy: *n* = 6953 age: 63.7 ± 7.5	1.5 T, Dixon fat-water	bilateral measurements, automatic segmentation: - atrophy: aCSA; - FI: IMF	Atrophy of all examined muscles in CLBP patients compared to healthy volunteers (*p* < 0.001) and no associations to acute LBP. Increased FI of all examined muscles in CLBP patients compared to healthy volunteers (*p* < 0.001) and acute LBP patients (*p* ≤ 0.044).
Giordan, 2023 [20]	MF, ES, PM, PPM	multilevel, L1–S1	CLBP *n* = 153 age: 58.5 ± 3.3, Healthy: *n* = 52 age: 49.5 ± 4.3	3 T, T1 + T2	bilateral measurements, semi-automatic - atrophy: CSA, TMV; - FI: FI	Atrophy (CSA) of MF (*p* = 0.004), ES (*p* = 0.032), and PM (*p* = 0.004) in CLBP patients compared to healthy volunteers. Atrophy (TMV) of PPM in CLBP patients compared to healthy volunteers (*p* < 0.0001). Increased FI (TMV) of PPM in CLBP patients compared to healthy volunteers (*p* < 0.0001).
Chen, 2024 [28]	MF, PPM	multilevel, L4–S1	CLBP *n* = 100 age: 52.1 ± 8.8 aLBP patients *n* = 120 age: 51.3 ± 9.7, Healthy *n* = 80 age: 53.2 ± 10.4	3 T, T1 + T2	segmentation - atrophy: tCSA, fCSA; - FI: Kjaer method and fatty degeneration	Atrophy of MF in CLBP patients compared to healthy volunteers (*p* = 0.023). Increased FI of PPM (*p* = 0.009) and of MF (*p* = 0.002) in CLBP patients compared to healthy volunteers.
Sions, 2017 [29]	MF, ES, PM, QL	multilevel, L2–L5	CLBP *n* = 53 age: 68.1 ± 71.7 Healthy *n* = 49 age: 70.3 ± 74.1	1.5 T, T1	bilateral measurements, manual segmentation: - atrophy: rCSA, rmCSA; - FI: MFI	Atrophy of ES in CLBP patients compared to healthy volunteers (*p* = 0.011). Increased FI of MF in CLBP patients compared to healthy volunteers (*p* = 0.016). No associations were found in PM and QL.
Mamatha, 2024 [21]	MF, ES, PM, QL	single level, L4 upper endplate	CLBP *n* = 50 age: 50 ± 10, Healthy *n* = 50 age: 50 ± 10	1.5 T, T2	bilateral measurements, semi-automatic - atrophy: CSA; - FI: Tissue Quant algorithm	Atrophy of MF and ES in CLBP patients compared to healthy volunteers (*p* = 0.000). Increased FI of MF and ES in CLBP patients compared to healthy volunteers. No associations were found in PM and QL.

MF—multifidus; ES—erector spinae; PM—psoas major; QL—quadratus lumborum; PPM—paraspinal muscles; LBP—low back pain patients; CLBP—chronic low back pain patients; CNLBP—chronic non-specific low back pain patients; LDH—lumbar disc herniation patients; LSI—lumbar spinal instability patients; Healthy—healthy volunteers; MRI—magnetic resonance imaging; T—Tesla; T1—T1 weighted image; T2—T2 weighted image; CSA—cross-sectional area; rCSA—relative cross-sectional area; rmCSA—relative muscle cross-sectional area; tCSA—total cross-sectional area; fCSA—functional cross-sectional area; mCSA—muscle cross-sectional area; aCSA—average cross-sectional area; TMV—total muscle volume; FF—fat fraction; FI—fat infiltration; IMF—intramuscular fat; PDFF—proton density fat fraction.

**Table 3:** Summary of findings in the selected studies of muscle atrophy and increased FI in lumbosacral muscles of CLBP patients.

Segmentation Method	Authors	Atrophy					Increased FI					1.5 T MRI Power (Tesla)	3 T MRI Power (Tesla)
		MF	ES	PM	QL	PPM	MF	ES	PM	QL	PPM		
Manual	Huang et al., 2022 [23]	**Yes**	**No**	N/A	N/A	N/A	**Yes**	**Yes**	N/A	N/A	N/A	No	**Yes**
	Fan et al., 2023 [25]	**Yes**	**Yes**	N/A	N/A	N/A	**Yes**	**Yes**	N/A	N/A	N/A	No	**Yes**
	Zhu et al., 2023 [27]	**No**	**No**	**No**	N/A	N/A	**Yes**	**Yes**	**Yes**	N/A	N/A	**Yes**	No
	Sions et al., 2017 [29]	**No**	**Yes**	**No**	**No**	N/A	**Yes**	**No**	**No**	**No**	N/A	**Yes**	No
Semi-automatic	Alami et al., 2024 [19]	**Yes**	**Yes**	**No**	N/A	N/A	**Yes**	**No**	**No**	N/A	N/A	**Yes**	No
	Giordan et al., 2023 [20]	**Yes**	**Yes**	**Yes**	N/A	**Yes**	N/A	N/A	N/A	N/A	**Yes**	No	**Yes**
	Mamatha et al., 2024 [21]	**Yes**	**Yes**	**No**	**No**	N/A	**Yes**	**Yes**	**No**	**No**	N/A	**Yes**	No
	Sions et al., 2016 [18]	**No**	N/A	N/A	N/A	N/A	**Yes**	N/A	N/A	N/A	N/A	**Yes**	No
Automatic	Wesselink et al., 2024 [22]	**Yes**	**Yes**	**Yes**	N/A	N/A	**Yes**	**Yes**	**Yes**	N/A	N/A	**Yes**	No
N/A	Chen et al., 2024 [28]	**Yes**	N/A	N/A	N/A	**No**	**Yes**	N/A	N/A	N/A	**Yes**	No	**Yes**
	Chen et al., 2023 [26]	**Yes**	N/A	**No**	N/A	N/A	**Yes**	N/A	**No**	N/A	N/A	No	**Yes**
	Sakai et al., 2017 [24]	**Yes**	**Yes**	N/A	N/A	N/A	**Yes**	N/A	N/A	N/A	N/A	N/A	N/A

FI—fatty infiltration; MF—multifidus; ES—erector spinae; PM—psoas major; QL—quadratus lumborum; PPM—paraspinal muscles; MRI—magnetic resonance imaging; T—Tesla; N/A—not applicable. Bold formatting was used for “Yes” and “No” responses to distinguish them from “N/A” entries.

## 4. Discussion

To the best of our knowledge, this is the first scoping review to map and summarize the literature on soft-tissue alterations in the lumbosacral area in CLBP patients following PRISMA-ScR guidelines [15]. The high heterogeneity of the included studies made direct comparisons difficult. Despite that, the available evidence suggests recurring patterns of muscle degeneration, specifically atrophy and increased FI, in lumbosacral muscles among patients with CLBP. However, these findings should be interpreted with caution, given the methodological variability among the included studies. Moreover, no studies reported evidence of muscle degeneration in healthy control groups, further supporting the association between structural muscle changes and CLBP. In addition, we found relationships between the technical aspects and outcomes of lumbosacral muscle assessment in CLBP.

### 4.1. Degeneration of Lumbosacral Muscles in CLBP Patients

Our scoping review identified soft-tissue changes in all examined muscles except QL. Increased FI was observed in all studies for at least one of the examined muscles. Only the studies carried out by Sions et al. [29] and Zhu et al. [27] showed no atrophy of the examined muscles (MF in both studies and ES and PM in the second study). Generally, the composition of the MF muscle seems to be associated with LBP, whereas no such association has been observed in ES and PM muscles [30]. Despite that, we noticed that all muscles were generally susceptible to both types of degeneration. MF appeared to be the most consistently affected muscle and commonly presented both atrophy and increased FI in CLBP patients [18,19,20,21,22,23,25,26,27,28,29], which aligns with previous studies [31]. However, ES also appears to be associated with a high susceptibility to atrophy and increased FI in CLBP patients [19,20,21,22,23,24,27,29]. PM showed moderate atrophy and increased FI when compared to MF and ES [20,22,27]. Moreover, when MF, ES, and PM were examined together as paraspinal muscles (PPM), they also presented both atrophy and increased FI [20], or at least increased FI when atrophy was not explored [23].

Degeneration changes in all PPM muscles, rather than only MF, probably occurred because we evaluated only CLBP patients or LBP patients meeting the chronic pain criteria, not all LBP patients, as was done before [30]. This suggests that, while MF changes might occur in all LBP patients, ES and PM alterations may be closely related to chronic pain. The absence of degeneration in QL might mean that it is somehow resistant to degeneration changes and/or is not affected by CLBP. Another possible explanation could be the limited inclusion of QL in imaging protocols.

The high heterogeneity of the studies prevented us from making muscle-specific comparisons of muscle degeneration trends in CLBP. However, based solely on atrophy and increased FI, this scoping review revealed a correlation between the examined factors of muscle degeneration. All cases of atrophy were accompanied by increased FI, indicating that muscle atrophy is often associated with increased FI. However, not all cases with increased FI showed signs of atrophy.

Altogether, these findings support the hypothesis of multifactorial and complex muscle deterioration changes in patients with CLBP [32,33]. They also indicate that soft-tissue assessment may be a promising direction for identifying correlates of CLBP. However, the current evidence is insufficient to validate these measures as clinical biomarkers of pain chronicity, severity, and functional limitations in this population. Our results also indicate that both muscle atrophy and increased FI should be examined together for better prognostic value. It also seems promising to study the paraspinal muscles as a single group. This approach facilitates analysis without the need to distinguish individual muscles, thereby accelerating the measurement process.

### 4.2. Technical Aspects of Lumbosacral Muscle Assessment in CLBP Patients

The technical aspects of lumbosacral muscle assessment in CLBP are an important issue. Most modern studies utilize MRI in CLBP research as it is the gold standard for soft-tissue assessment [34], which is why our review is based exclusively on this imaging technique. Image segmentation enables computer-aided diagnostics and is a key component of intelligent medicine, enhancing diagnostic precision and reliability [35]. It allows precise determination of shape, size, volume, and extraction of desired anatomical structures. These factors are especially important for lumbosacral muscles, which are characterized by heterogeneous FI and difficulty in distinguishing anatomical edges between them [36,37]. This may explain why some studies in this review did not show morphological muscle changes while others did.

This review analyzed changes in lumbosacral muscle degeneration at different anatomical levels or ranges of levels. The level at which the measurements are conducted may be crucial for detecting muscle degeneration in CSA assessment. Several studies examining muscles at multiple levels revealed that muscle atrophy and/or increased FI only occurred at some of the examined levels [38,39,40]. To avoid errors, future studies using CSA analysis should conduct measurements in a designated area [36,41].

The volume of assessed muscle is likely to cause differences in the precision and consistency of the results obtained in patients with CLBP. In this review, only one study utilized volumetric measurement [20]. This may be explained by the methodological requirements of volumetric analysis. Reliable volumetric reconstruction requires MRI acquisitions with sufficient anatomical coverage and multiple contiguous slices [42]. Moreover, TMV assessment is considerably more time-consuming than CSA measurements because it requires segmentation of the entire muscle across multiple consecutive MRI slices [41]. In comparison, CSA measurements are obtained from a single slice at a predefined vertebral level. Therefore, CSA represents only a localized estimate of muscle morphology [43]. Given that muscle degeneration may occur unevenly along the muscle length, single-slice CSA measurements may not fully capture the overall extent of muscle loss, compared to TMV measurements [44]. Consequently, some studies using CSA at various anatomical levels reported different degrees of atrophy depending on the examined anatomical level [19,22,23,27]. Although direct comparisons between CSA and TMV remain limited, TMV may provide a more comprehensive assessment of muscle degeneration by evaluating the entire muscle volume. Nevertheless, current evidence is insufficient to determine whether TMV is superior to CSA, and it would be beneficial to perform both CSA and TMV assessments on a cohort to directly compare their effectiveness in evaluating CLBP. Furthermore, advancements in automated image analysis may reduce the time burden associated with volumetric assessment, facilitate three-dimensional muscle reconstruction and improve the utilization of large imaging datasets [45].

It is also crucial to consider how muscle CSA and FI are selected and defined. Studies in this review employ various approaches to muscle assessment. Atrophy was measured using CSA, rCSA, rmCSA, tCSA, fCSA, mCSA, aCSA, TMV, whereas increased fatty infiltration was measured using FF, FI, IMF, PDFF and the Kjaer method. Those parameters reflect different aspects of muscle morphology and composition and are characterized by various methodologies that can influence the study’s outcomes. For example, CSA-based measurements reflect overall muscle size, while functional CSA specifically quantifies the contractile muscle component [19,29]. Consequently, CSA measurements may underestimate the extent of muscle degeneration in muscles with substantial fatty infiltration. Similarly, the differences in the assessment of fatty infiltration may contribute to variability between studies. For example, semiquantitative grading systems, such as the Kjaer classification, provide categorical estimates of fat content and may be less sensitive to subtle differences between individuals [46]. In contrast, quantitative measures such as PDFF allow a more precise evaluation of intramuscular fat accumulation [47]. Consequently, the use of semiquantitative grading systems may contribute to differences in the reported extent of fatty infiltration when compared with quantitative MRI-based assessments. To enable future utilization of this method as a diagnostic biomarker for CLBP, this should be further addressed.

In reviewed studies, MRI systems with field strengths of 1.5 T and 3.0 T were used. Generally, a 3 T field strength is more precise for soft-tissue imaging than 1.5 T MRI, due to its higher signal-to-noise ratio and enhanced image contrast [48,49]. This is consistent with our results. 3 T MRI appears to provide more consistent and reliable detection of both atrophy and increased FI, with no cases missed across studies, as in 1.5 T MRI. However, due to the substantial variability in the examined studies, we cannot determine which acquisition method will be most adequate for this type of analysis. Moreover, differences in MRI sequences may also influence the assessment of fatty infiltration. The included studies used a variety of MRI sequences, such as T1, T2, and Dixon-based acquisitions. So far, both T1- and T2-weighted MRI have been widely used for assessment of morphological muscle changes in the lumbosacral area [36]. However, chemical-shift MRI techniques, such as Dixon-based techniques that enable separation of fat and water signals, may allow a more quantitative and reproducible assessment of intramuscular fat content [36,50]. Therefore, differences in MRI sequence selection may contribute to heterogeneity across studies and should be considered when interpreting and comparing findings.

Moreover, variability in segmentation methods may also influence these findings. Manual segmentation is considered the most precise method for determining muscle volume and CSA [51]. However, our study revealed that both manual and semi-automatic segmentation presented comparable performance in identifying morphological changes in lumbosacral muscles. Although both can be utilized for soft-tissue assessment in CLBP patients, the semi-automatic method enables quicker measurements and may therefore be more promising for future research. Additionally, although automatic segmentation is the fastest method, it currently yields the lowest detection rates in soft-tissue assessment [52]. Although the automatic method was effective in detecting muscle degenerative changes in this scoping review, we were able to assess only one study. All this highlights the importance of selecting the appropriate technique for accurate assessment of muscle degeneration. Based on the mapped evidence, 3 T MRI and semi-automatic segmentation appear to be promising approaches for future studies. Nevertheless, direct comparisons of these approaches are needed before any can be recommended as superior.

### 4.3. Limitations and Future Directions

The main limitation of this research was the high heterogeneity among included studies. This forced us to focus solely on the overall outcome of the study, preventing us from identifying muscle-specific degeneration trends and direct correlations between them. Moreover, we identified several technical factors that can influence the consistency of the results in this diagnostic method for CLBP. Firstly, choosing different anatomical levels or ranges of levels for segmentation. Secondly, the MRI acquisition method and the choice of a segmentation method. Thirdly, the accuracy and execution of TVM and CSA measurements. Lastly, how muscle atrophy and increased FI are defined and measured. These factors may contribute to the inconsistency or power significance of results and should be addressed in future studies.

Another limitation of this review is the application of strict eligibility criteria. While this approach helped maintain the focus of the review and improved the comparability of the included studies, it may also have reduced the amount of available evidence. Some studies were excluded because they did not use segmentation of MRI images, assessed different muscle outcomes, or included comparison groups other than healthy controls. Therefore, the findings should be interpreted as a focused overview of MRI-segmentation-based evidence, rather than a comprehensive summary of all research on soft-tissue changes in CLBP.

Additionally, the studies encompassed highly heterogeneous populations, with sample sizes ranging from 30 to 1681 for CLBP patients and from 15 to 6953 for healthy controls. Only a few studies included alternative comparison groups, such as patients with acute LBP or asymptomatic individuals with lumbar disc herniation, limiting the ability to differentiate degeneration specific to CLBP. Therefore, it is crucial to include larger and more diverse populations of CLBP patients in future studies.

Furthermore, this review also revealed the correlation between muscle atrophy and increased FI. Therefore, it may be useful to establish coefficients for atrophy and increased FI, rather than examining them independently.

Finally, future studies should also explore the impact of clinical factors on muscle degeneration changes in CLBP. CLBP patients are a heterogeneous group [53], and we suspect that not all patients will present the same degree of muscle degeneration changes. This might be related to differences in age, pain duration, and pain intensity levels [54,55,56,57,58]. Moreover, different pain mechanisms can be involved in chronic pain [59]. Future identification of specific patient subpopulations based on degenerative muscle changes can contribute to better therapeutic outcomes for CLBP patients. It is also important to investigate whether morphological muscle changes are a cause or an effect of CLBP. Longitudinal studies are necessary to address this issue.

## 5. Conclusions

Assessment of soft tissue in the lumbosacral area may provide a useful objective information tool for CLBP patients. Despite differences in study methodology, all examined muscles, except the quadratus lumborum, showed degenerative changes to some extent. The MF muscle is generally the most affected. Alterations of ES and PM muscles were also observed in association with CLBP, as they usually do not present degenerative changes in acute LBP. QL seems to be less affected by these changes. A correlation between soft-tissue alterations, such as muscle atrophy and increased FI, suggests that, in the future, these factors may represent potential objective biomarkers for chronic pain, severity, and functional limitations in CLBP patients. However, their clinical relevance remains to be established. It can be useful for further studies to establish coefficients for atrophy and increased FI, rather than examining them independently. Moreover, the choice of MRI acquisition and segmentation methods may also influence the assessment of muscle morphology. Based on the mapped evidence, the 3 T field strength appears to be more precise for soft tissue imaging than 1.5 T MRI, and semi-automatic segmentation may be the most appropriate method for this type of analysis. However, direct comparisons between these methods are needed before definitive conclusions can be drawn. Consequently, this review should be viewed as a focused synthesis of the current MRI segmentation-based evidence, rather than as clinical validation of a specific biomarker.

## Figures and Tables

**Table 1:** Search strategy in multiple databases.

Database	Keywords	Results	Date
PubMed	(LBP OR Low back pain OR (Back Pain AND Low) OR (Pain AND Low Back) OR Lower Back Pain OR (Back Pain AND Lower) OR (Pain AND Lower Back)) AND (muscle size OR muscular size OR cross sectional area OR csa OR muscular size OR muscle thickness OR muscular thickness OR muscular atrophy OR (atrophy AND muscular) OR (atrophy AND muscle) OR muscle atrophy OR muscle degeneration OR fat infiltration OR fatty infiltration OR fat deposition OR intramuscular fat OR fat tissue OR fatty tissue OR adipose tissue OR fat OR muscle structure OR muscular structure OR muscle morphology OR (morphology And muscle) OR muscular morphology OR muscle composition OR muscular composition OR soft tissue morphology OR soft tissue composition OR segmentation OR volumetric assessment OR muscle volume OR 3D assessment OR muscle imaging OR quantitative imaging)	1136	15 October 2024
Scopus	TITLE-ABS (“LBP” OR “Low back pain” OR “(Back Pain AND Low)” OR “(Pain AND Low Back)” OR “Lower Back Pain” OR “(Back Pain AND Lower)” OR “(Pain AND Lower Back)” AND “muscle size” …)	1722	15 October 2024
Embase	(lbp:ab,ti OR ‘low back pain’:ab,ti OR (‘back pain’:ab,ti AND low:ab,ti) OR (pain:ab,ti AND ‘low back’:ab,ti) OR ‘lower back pain’:ab,ti OR (‘back pain’:ab,ti AND lower:ab,ti) OR (pain:ab,ti AND ‘lower back’:ab,ti)) AND (‘muscle size’:ab,ti …)	1633	15 October 2024
Web of Science	(LBP OR Low back pain OR (Back Pain AND Low) OR (Pain AND Low Back) OR Lower Back Pain OR (Back Pain AND Lower) OR (Pain AND Lower Back)) AND (muscle size …) (Title) or (LBP OR Low back pain OR (Back Pain AND Low) OR (Pain AND Low Back) OR Lower Back Pain OR (Back Pain AND Lower) OR (Pain AND Lower Back)) AND (muscle size …) (Abstract)	2760	15 October 2024

## Data Availability

The original contributions presented in this study are included in the article/Supplementary Material. Further inquiries can be directed to the corresponding author.

## Supplementary Materials

The following supporting information can be downloaded at: https://www.mdpi.com/article/10.3390/diagnostics16121832/s1, Supplementary File S1: Preferred Reporting Items for Systematic reviews and Meta-Analyses extension for Scoping Reviews (PRISMA-ScR) Checklist. Table S1. Clinical diagnostic criteria, pain duration, assessment, and additional imaging parameters for the CLBP patients.

## Abbreviations

The following abbreviations are used in this manuscript:

aCSA	Average cross-sectional area
CLBP	Chronic low back pain
CNLBP	Chronic non-specific low back pain
CSA	Cross-sectional area
ES	Erector spinae
fCSA	Functional cross-sectional area
FF	Fat fraction
FI	Fatty infiltration
IMF	Intramuscular fat
LBP	Low back pain
LDH	Lumbar disc herniation
LSI	Lumbar spinal instability
mCSA	Muscle cross-sectional area
MF	Multifidus
MRI	Magnetic resonance imaging
PDFF	Proton density fat fraction
PM	Psoas major
PPM	Paraspinal muscles
QL	Quadratus lumborum
rCSA	Relative cross-sectional area
rmCSA	Relative muscle cross-sectional area
T	Tesla
tCSA	Total cross-sectional area
TMV	Total muscle volume
