# Supplementary material for: Magnetic Resonance Imaging Segmentation of Soft Tissue in the Diagnosis of Chronic Low Back Pain: A Scoping Review
Source: Diagnostics (Basel). 2026 Jun 13;16(12):1832. doi: 10.3390/diagnostics16121832 (PMC13298480; doi:10.3390/diagnostics16121832)
Supplement: Supplementary file 1 [file diagnostics-16-01832-s001.zip › Table S1 (suplementary table).pdf]

Supplementary Table S1. Clinical diagnostic criteria, pain duration, assessment, and additional imaging parameters for the CLBP patients.

	Description of chronic pain population	Pain duration	Pain assessment	Pain location	Disability assessment	Functional assessment	Additional imaging parameters
Sions, 2016 [18]	Community-dwelling older adults aged 60–85 years with CLBP	≥3 months	Pain intensity ≥3/10	LBP	N/A	Short Form-36 Health Survey, The Timed Up and Go, gait speed, fast stair descent	N/A
Huang, 2022 [23]	Untreated patients with CLBP	>3 months	VAS = 5.99 ± 1.42	LBP	ODI = 28.45 ± 13.17	N/A	N/A
Sakai, 2017 [24]	Patients aged ≥65 years with CLBP and no lower-extremity symptoms	>3 months prior to treatment	VAS = 6.5 ± 2.1	LBP	RDQ = 16.9 ± 7.9	Isometric trunk muscle strength: back muscle strength and abdominal muscle strength	Lumbar scoliosis angle, lumbar lordosis angle, sacral inclination angle, spondylolisthesis, lumbar range of motion
Fan, 2023 [25]	Patients aged 10–40 years with CLBP	≥3 months	VAS = 6.55 ± 1.25	LBP	N/A	N/A	Lumbar lordosis, annulus fibrosus tear, lumbar back fasciitis, Pfirrmann grade, Modic changes
Chen, 2023 [26]	Patients aged ≥18 years diagnosed with LBP with LDH	>12 weeks, mean: 22.4 ± 6.42	VAS LBP = 7.64 ± 1.77	LBP and leg pain	ODI = 26.8 ± 9.72	N/A	Pfirrmann grade

			VAS leg pain = 7.43 ± 1.80				
Zhu, 2023 [27]	Female nurses aged 20–40 years CBNLBP	>3 months (Methods) / >12 months (Table 1)	N/A	Pain below T12 and above the buttocks line	N/A	N/A	N/A
Alami, 2024 [19]	Patients aged 18–45 years with CNLBP with LSI or without LSI	>3 months, persistent or recurrent with at least 6 months elapsed since the first episode	VAS CNLBP with LSI = 4.7 ± 1.68,<br><br>VAS CNLBP without LSI = 4.8 ± 2.43	LBP	ODI CNLBP with Clinical LSI = 31.67 ± 9.00; ODI CNLBP without Clinical LSI = 30.53 ± 12.38	LSI group: negative SLR, ≥1 positive aberrant movement pattern, positive prone instability test	N/A
Wesselink, 2024 [22]	Participants from the UK Biobank with CBP	>3 months	N/A	BP	N/A	IPAQ	N/A
Giordan, 2023 [20]	Adults aged 18–65 years with BP: degenerative disc disease alone or with degenerative lumbar stenosis, degenerative spondylolisthesis, disc degeneration,	>6 months	VAS = 6.8 ± 1.8	Lumbar back pain (LBP, buttocks and leg pain)	ODI = 45.2 ± 16.1	N/A	Disc height, Pfirrmann grade, Modic changes, lumbar spinal stenosis, degenerative spondylolisthesis, pelvic incidence, pelvic tilt, lumbar

	and/ or Modic changes;						lordosis, PI–LL mismatch
Chen, 2024 [28]	CLBP with LDH undergoing discectomy; all patients had unsuccessful conservative treatment >12 weeks	≥12 weeks	LBP VAS = 6.78 ± 2.02;<br>leg pain VAS = 7.11 ± 1.45	LBP and leg pain	ODI = 24.77 ± 7.22	N/A	Pfirrmann grade, Modic changes, facet fluid sign
Sions, 2017 [29]	Older adults aged 60-85 years with nonspecific CLBP	>3 months; at least 4 days/week	NRS ≥ 3/10; NRS LBP = 3.5 (95% CI 3.0-3.9)	LBP	Modified ODI = 33.4% (95% CI 30.5-36.4)	N/A	N/A
Mamatha, 2024 [21]	Male patients aged 40-60 years with bilateral CBP	≥3 months; at least 4 days/week	NRS; moderate intensity ≥3/10	bilateral LBP	N/A	N/A	IVD abnormalities (standard alignment/bulge/ protrusion/extrusion), Modic type I changes

Legend: CLBP - chronic low back pain; LBP - low back pain; N/A - not applicable; VAS - visual analoge scale -VAS; ODI - Oswestry Disability Index; MRI - magnetic resonance imaging; RDQ - Roland Morris Disability Questionnaire; LDH - lumbar disc herniation; CBNLBP - chronic bilateral non-specific low back pain; CNLBP - chronic non-specific low back pain; LSI -Lumbar spinal instability; SLR - straight leg rise test; UK - United Kingdom; CBP - Chronic Back Pain; IPAQ - Short International Physical Activity Questionnaire; CNN - computer neural network; BMI - body mass index; FOV - field of view; PI - pelvic incidence; LL - Lumbar lordosis; LDH - lumbar disc herniation; NRS - numeric rating scale; IVD - intervertebral disc
